# Salivary MMP‐13 gender differences in periodontitis: A cross‐sectional study from Sweden

**DOI:** 10.1002/cre2.76

**Published:** 2017-09-15

**Authors:** Eunice Virtanen, Maha Yakob, Taina Tervahartiala, Per‐Östen Söder, Leif C. Andersson, Timo Sorsa, Jukka H. Meurman, Birgitta Söder

**Affiliations:** ^1^ Department of Oral and Maxillofacial Diseases University of Helsinki and Helsinki University Hospital Finland; ^2^ Department of Dental Medicine Karolinska Institutet Sweden; ^3^ Department of Pathology, Haartman Institute University of Helsinki Finland

**Keywords:** biomarkers, saliva, periodontitis, matrix metalloproteinase 13

## Abstract

We investigated serum and saliva concentrations of matrix metalloproteinases, MMP‐8, MMP‐9, and MMP‐13, and their tissue inhibitor TIMP‐1, in a group of patients with and without periodontitis from Sweden. The hypothesis was that these biomarkers are higher in the periodontitis patients. Ninety patients participated in this cross‐sectional study. Fifty‐one patients had periodontitis whereas 39 were periodontally healthy. Saliva and serum samples were analyzed with immunofluorometric, enzyme‐linked immunosorbent assay and western blot. Results were statistically analyzed with independent t test, Mann–Whitney U test, Bonferroni corrections, and regression analyses. MMP‐13 was not detected in serum, but in saliva, higher values were found among the periodontally healthy compared with periodontitis subjects (0.32 ± 0.26 vs. 0.21 ± 0.23 ng/ml, p < .05). Female gender and clinical attachment loss were the explanatory factors for higher salivary MMP‐13 values with odds ratio 3.08 (95% confidence interval [1.17, 8.11]) and 3.57 (95% confidence interval [1.08, 11.82]), respectively. No statistically significant differences between groups were found in serum and saliva values of MMP‐8, MMP‐9, and TIMP‐1. Contrary to our hypothesis, no statistically significant differences between patients with and without periodontitis were seen in MMP‐8, MMP‐9, and TIMP‐1 values. However, higher MMP‐13 concentrations in saliva were associated with female gender and higher clinical attachment loss. Metabolism of MMP‐13 may thus have some gender implications in periodontitis.

## 1 INTRODUCTION

Matrix metalloproteinases (MMPs) are important molecules in physiological processes such as reproduction, embryonic development, and tissue remodeling. MMPs are also involved in the pathological processes of periodontitis (Gursoy et al., 2013; Sorsa et al., 2006), in other inflammatory diseases (Clutterbuck, Asplin, Harris, Allaway, & Mobasheri, 2009), and even in cancer (Hadler‐Olsen, Winberg, & Uhlin‐Hansen, 2013). MMPs can promote surrogate tissue destruction in diseases by degrading almost all extracellular proteins. Additionally, MMPs can process various nonmatrix bioactive substrates such as proinflammatory and anti‐inflammatory cytokines, chemokines, growth factors, serpins, serum components, complement components, and cell signaling molecules, thereby modifying immune responses (Khokha, Murthy, & Weiss, 2013). Furthermore, MMPs can activate each other in cascades (Dufour & Overall, 2013; Hadler‐Olsen et al., 2013; Khokha et al., 2013; Sorsa et al., 2006). Thus, the outcome of MMP actions is not only surrogate for tissue destruction but may also be anti‐inflammatory or protective (Dufour & Overall, 2013).

MMP‐8, MMP‐9, and their metalloproteinase inhibitor‐1 (TIMP‐1) have been particularly investigated as periodontitis markers (Gorska & Nedzi‐Gora, 2006). They have been extensively studied from oral fluids (Gursoy et al., 2010; Gursoy et al., 2013). MMP‐8 mostly acts in the metabolism of collagen type I, the most common collagen in the periodontal ligament, whereas MMP‐9, a type IV collagenase and gelatinase B, is involved in basement membrane remodeling. It can be produced by periodontal ligament fibroblasts and polymorphonuclear cells by inflammation, and it is responsible for type IV collagen degradation at the sulcus epithelium and gingival connective tissue (Chang, Yang, Lai, Liu, & Hsieh, 2002).

MMP‐13 (collagenase 3) expression was first discovered in breast cancer (Freije et al., 1994). It has been particularly involved in inflammatory diseases such as rheumatoid arthritis and osteoarthritis where it associates with resorption and destruction of bone and cartilage (Goldring et al., 2011). It is expressed by different cells of the periodontium and inflammatory cells in association with chronic periodontitis (CP; Hernandez et al., 2006; Hernandez Rios et al., 2009). MMP‐13 levels have been higher in gingival crevicular fluid of CP patients relative to healthy subjects (Hernandez, Martinez, Tejerina, Valenzuela, & Gamonal, 2007). In saliva, MMP‐13 has been augmented in localized periodontitis but decreased in generalized periodontitis (Gursoy et al., 2013).

TIMP‐1 is an endogenous inhibitor of MMPs implicated in inflammation and cancer processes. The balance between MMPs and TIMPs is important in the degradation of extracellular matrix (Nagase, Visse, & Murphy, 2006).

On the basis of this background, the aim of our present study was to compare the levels of MMP‐13, MMP‐9, MMP‐8, and TIMP‐1 in saliva and serum and to analyze possible associations of the MMPs with the periodontal status of our subjects. Our study hypothesis was that patients with periodontitis have higher levels of these biomarkers in saliva and serum comparted with those with no periodontitis. This might possibly reflect higher infection/inflammation both locally in the mouth and systemically in the circulation.

## 2 MATERIALS AND METHODS

### 2.1 Study population

**Figure 1:**
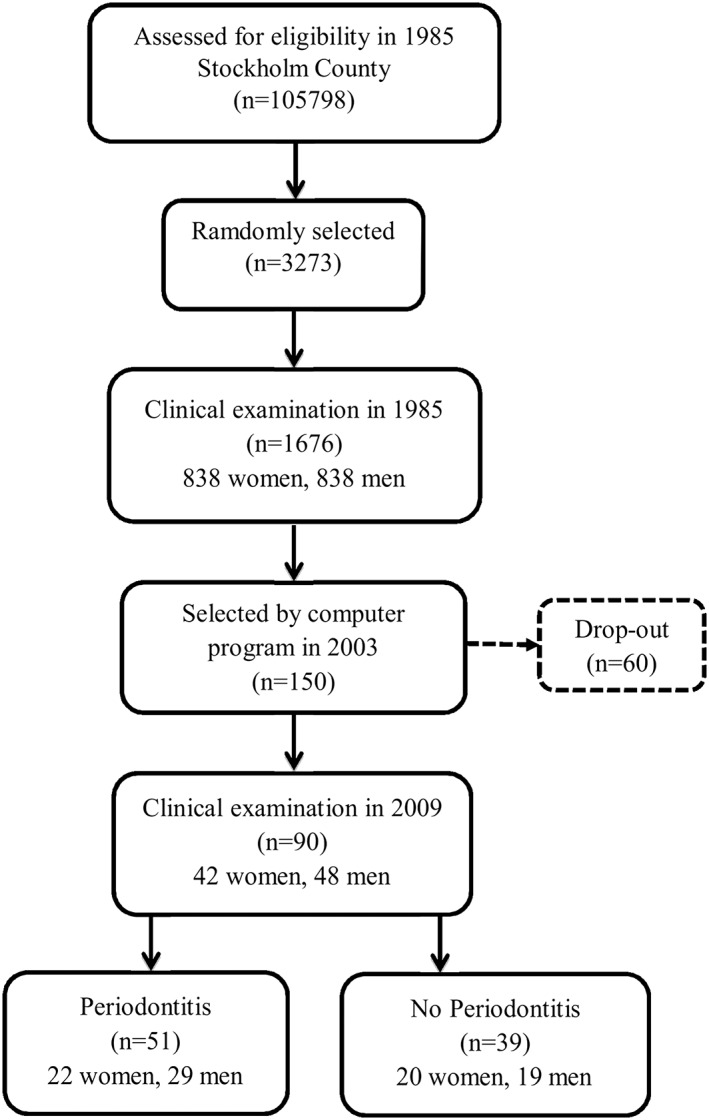
Study population flow chart

The study population is based on a random sample cohort representative of the inhabitants of the Stockholm County, born on the 20th of any month from 1945 to 1954; the number of eligible patients was 105,798, and from these, 3,273 individuals were randomly selected for the first study in the year 1985. Of them, 1,676 were clinically examined with particular focus on periodontal disease (P. O. Soder, Jin, Soder, & Wikner, 1994). In 2001, age and gender matched subjects were selected from the original cohort with a computer program from the two clinical groups, subjects with periodontitis (*n* = 100) and without periodontitis (*n* = 50). In 2009, these subjects were recalled for a further follow‐up and 90, then 54–64 years old, participated in our present study (Figure 1). Details of the study group have been earlier reported (Yakob et al., 2012). Our present study is a cross‐sectional investigation because no biomarker data were collected at baseline in 1985.

### 2.2 Ethical consideration

The Regional Ethical Review Board of Stockholm, situated at the Karolinska Institutet and Huddinge University Hospital, Sweden, had approved the study protocol (Dnr 101/85 and Dnr 2007/1669‐31). The study is in accordance with the 1975 Declaration of Helsinki, as revised in 1983. All subjects gave their written informed consent to participate in the study.

### 2.3 Clinical examination and demographic data

The clinical examination and sampling in 2009 was made by one of the authors (M. Y.) who at that time was a PhD student at the Department of Dental Medicine in Karolinska Institutet. Gingival inflammation was recorded for all the remaining teeth using the gingival index (Löe and Silness, 1963), bleeding on probing (BOP) as a marker of inflammation (Lang et al., 2000), and oral hygiene status using the plaque index (Silness & Loe, 1964), and calculus index (Greene & Vermillion, 1964). Probing depth and clinical attachment loss (CAL) for each tooth were assessed from six surfaces using a periodontal probe (Hu‐Friedy^®^PCPUNK 15, Chicago, IL, USA). The number of the remaining teeth was also registered. Basic demographic data such as age, gender, and smoking habits (nonsmokers and smokers/ex‐smokers/snuff users) were recorded at the time of the clinical examination. In the periodontitis classification, the criteria used were one or more deep pockets ≥5 mm and BOP, according to Yakob et al. (2012) and B. Soder, Jin, Klinge, and Soder (2007).

### 2.4 Sampling and analyses of saliva for MMP‐8, MMP‐9, MMP‐13, and TIMP‐1

Patients were not allowed to eat, drink, or smoke for 1 hr before saliva sampling. Stimulated saliva was collected for 5 min by chewing a 1‐g piece of paraffin wax. Patients with dentures kept them in the mouth during the chewing. The amount of saliva collected was measured using graduated test tubes. All saliva samples were immediately deep frozen and stored for analyses (−75 °C). The MMP‐8 was analyzed with immunofluorometric assay (IFMA), and MMP‐9, MMP‐13, and TIMP‐1 were analyzed with enzyme‐linked immunosorbent assay (ELISA) specific for each biomarker, as in previous studies (Gursoy et al., 2010; Gursoy et al., 2013; Hernandez et al., 2006; Hernandez et al., 2007; Hernandez Rios et al., 2009). Molar ratio was calculated for MMP‐8, MMP‐9, and MMP‐13 to the inhibitor TIMP‐1. In addition, for the MMP‐13, a western blot analysis for each patient group was also made by selecting three representative patients from each group (two women and one man with MMP‐13 concentration in saliva ≥0,50 ng/ml, respectively). Recombinant human proMMP‐13 (Proteaimmun GmbH, Berlin, Germany) was used as positive control (Hernandez et al., 2007).

### 2.5 Sampling and analyses of blood

Antecubital venous blood samples were taken after 12‐hr overnight fasting at the Laboratory of Clinical Chemistry of the Karolinska University Hospital, Huddinge, Sweden. Serum samples were prepared and stored deep frozen for later analyses. Analyses of each biomarker were made using commercial specific ELISA assays for human MMP‐9, MMP‐13, and TIMP‐1 by following the instructions of the manufacturers. IFMA was used to analyze the MMP‐8, according to (Yildirim et al., 2013).

### 2.6 Statistical analyses

Descriptive statistics (mean and standard deviations) and other statistical analyses were made using the IBM SPSS Statistics program version 22. Independent *t* test for equality of means was used to compare the groups, and in case of obvious nonnormality, Mann–Whitney U test was used. The statistical significance was set at 0.05, two‐tailed. Bonferroni corrections for multiple comparisons were used. A logistic regression analysis with a backwards likelihood ratio method to eliminate insignificant variables was conducted, with a dichotomized measure of MMP‐13 in saliva as the dependent variable (a cutting value was more than 50% of the cumulative percentage) and all periodontal markers (BOP, CAL, gingival index, calculus index, plaque index, and probing depth) as explanatory variables and controlled for age, gender, and smoking habits. No test of normality or calculation of the sample size was made.

## 3 RESULTS

From the 90 patients included in the study at the 2009 follow‐up examination, 57% had periodontitis whereas 43% had no periodontitis (Figure 1). No significant differences between the groups were found in age, gender, and smoking habits (Table 1). However, as expected, all clinical markers of oral health were significantly higher in the periodontitis group, and the result was the same even after the Bonferroni correction for multiple comparisons (*p* < .05).

**Table 1:** Demographic, clinical, and biomarker data from 90 patients, reexamined in 2009, with respect to periodontitis

	Periodontitis (*n* = 51)	No periodontitis (*n* = 39)	*p* a
	Number, mean ± SD	Number, mean ± SD	
Age (years)	60.20 ± 2.99	59.00 ± 2.84	NS
Gender (women/men)	22/29	20/19	NS
Smoking (yes/no)	29/22	15/24	NS
**Gingival Index**	**0.93 ± 0.46**	**0.51 ± 0.26**	**<.001**
**Plaque Index**	**0.80 ± 0.40**	**0.51 ± 0.27**	**<.001**
**Calculus Index**	**0.39 ± 0.41**	**0.26 ± 0.21**	**.048**
**Bleeding on probing**	**0.58 ± 0.22**	**0.39 ± 0.17**	**<.001**
**Clinical attachment loss (mm)**	**3.54 ± 0.78**	**2.88 ± 0.46**	**<.001**
**Probing depth (mm)**	**3.09 ± 0.73**	**2.34 ± 0.26**	**<.001**
**No. of remaining teeth**	**25.76 ± 2.84**	**26.82 ± 1.63**	**.029**
MMP‐8 serum (ng/ml)	31.64 ± 26.56	35.88 ± 30.62	NS
MMP‐9 serum (ng/ml)	142.67 ± 99.38	165.51 ± 77.94	NS
MMP‐13 serum (ng/ml)	0.01 ± 0.06	0.00 ± 0.00	NS
TIMP‐1 serum (ng/ml)	142.24 ± 54.37	148.77 ± 46.17	NS
MMP‐8/TIMP‐1 serum (molar ratio)	0.82 ± 5.15	0.12 ± 0.14	NS
MMP‐9/TIMP‐1 serum (molar ratio)	2.67 ± 16.66	0.38 ± 0.24	NS
MMP‐8 saliva (ng/ml)	318.25 ± 87.98	301.84 ± 101.07	NS
MMP‐9 saliva (ng/ml)	189.87 ± 112.21	153.75 ± 105.10	NS
**MMP‐13 saliva (ng/ml)**	**0.21 ± 0.23**	**0.33 ± 0.26**	**.032**
TIMP‐1 saliva (ng/ml)	218.90 ± 113.59	218.25 ± 129.81	NS
MMP‐8/TIMP‐1 (molar ratio)	0.82 ± 0.54	0.76 ± 0.47	NS
MMP‐9/TIMP‐1 (molar ratio)	0.38 ± 0.40	0.31 ± 0.39	NS
**MMP‐13/TIMP‐1 saliva (molar ratio)**	**0.0005 ± 0.0006**	**0.0009 ± 0.0008**	**.038**

a *t* test for equality of means and Bonferroni corrections for multiple comparisons, **significance 2‐tailed**, and NS *p* > .05. Data are expressed as mean ± standard deviation (*SD*).

In serum, the MMP‐8, MMP‐9, and TIMP‐1 concentrations were slightly higher in the no‐periodontitis patients but the differences were not statistically significant. MMP‐13, however, was almost undetectable in serum; very low concentrations were registered particularly in the periodontitis patients. Due to these very low values of MMP‐13, it was not possible to calculate the molar ratio with TIMP‐1 from the serum samples.

**Figure 2:**
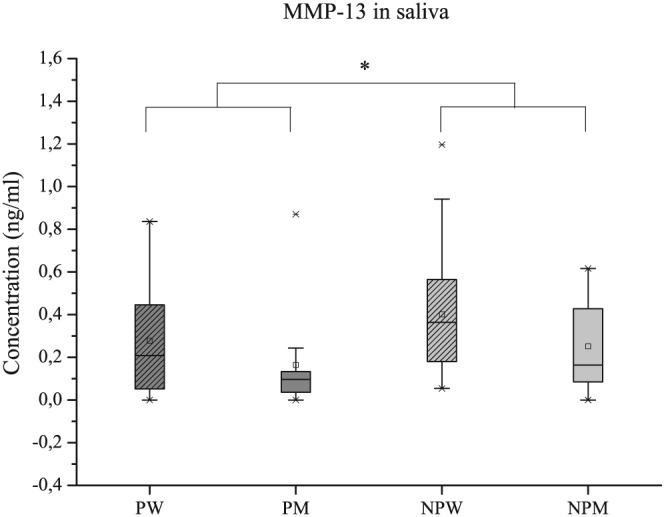
Distribution by gender of MMP‐13 in saliva in periodontitis and no periodontitis patients: women with periodontitis (PW), men with periodontitis (PM), women with no periodontitis (NPW), and men with no periodontitis (NPM). *p* < .05

In saliva, MMP‐8, MMP‐9, and TIMP‐1 values were slightly higher in the periodontitis patients, but again, the differences between groups were not significant. Corresponding findings were also recorded for MMP‐8/TIMP‐1 and MMP‐9/TIMP‐1 ratios (Table 1). But MMP‐13 values were significantly higher in the no‐periodontitis group when compared with the periodontitis patients (*p* = .032). The molar ratio of MMP‐13/TIMP‐1 further confirmed this result (*p* = .038; Table 1). The distribution of MMP‐13 concentrations in saliva among the periodontitis and no periodontitis patients are given in more detail in Figure 2.

**Figure 3:**
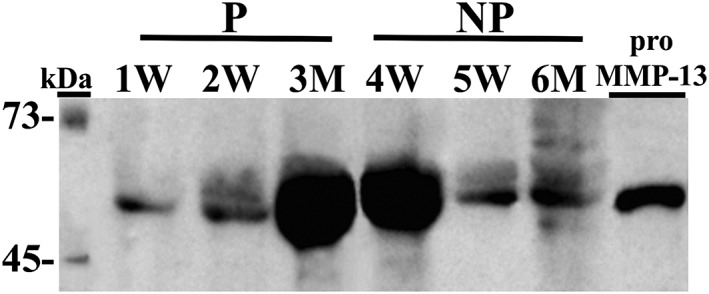
Molecular forms of salivary MMP‐13 assessed by western immunoblot on three representative samples for each group: periodontitis (P) and no periodontitis (NP) groups and women (W) and men (M). Recombinant human proMMP‐13 was used as positive control (on the right). Mobility at the molecular weight standard is given on the left

The MMP‐13 western immunoblots from the stimulated salivary samples revealed the immunoreactive bands corresponding to proMMP‐13 (60 kDa) in all representative samples. The predominant immunoreactivity for MMP‐13 was the 60‐kDa proform of MMP‐13 as shown in Figure 3. Densitometric quantification of the western immunoblot confirmed the presence of the protein in saliva.

In logistic regression analysis (controlled for age, gender, and smoking habits), the explanatory factors for the higher presence of MMP‐13 in saliva were female gender and CAL. The results are given in Table 2.

**Table 2:** Logistic regression analysis between the dependent variable “MMP‐13 in saliva” and several independent variables: age, gender, smoking habits, BOP, CAL, GI, CI, PLI and PD

Dependent variable	Explanatory variable	Beta	Chi‐square	*p* value	OR (95% confidence interval [CI])
	Female gender	1.13	5.18	.023	3.08 (95% CI [1.17, 8.11])
MMP‐13 saliva					
	CAL	1.27	4.35	.037	3.57 (95% CI [1.08, 11.82])

*Note*. Cox and Snell R^2^ = 0.18; Nagelkerke R^2^ = 0.24. BOP = bleeding on probing; CAL = clinical attachment loss; CI = calculus index; GI = gingival index; OR = odds ratio; PD = probing depth; PLI = plaque index. Please see the text for explanation.

## 4 DISCUSSION

We investigated how specific MMPs reflect in saliva and serum in patients with and without periodontitis in a sample from a large and well‐characterized study population from a Stockholm County cohort. As expected, the two groups were clearly different regarding the clinical periodontal markers (Table 1).

The main findings were that increased MMP‐13 concentration in saliva was associated with female gender and high CAL values. In the periodontal status classification we used, the “diseased” were patients with active periodontal inflammation reflected in BOP, instead of only recording deep pockets and attachment loss. Nevertheless, if MMP‐13 is related to bone and cartilage resorption, higher salivary values in patients with high CAL are understandable.

The finding with respect to gender difference is interesting because MMP‐13 was originally detected from breast cancer samples (Freije et al., 1994). It also seems to be involved in the migration of the breast cancer cells (Xue, Chen, Gu, Zhang, & Zhang, 2016). Breast cancer is known to principally affect women. Recent studies have shown a clear gender association between reduced bone stiffness in women and CAL (Silveira et al., 2016). In postmenopausal women with osteoporosis or osteopenia, higher CAL values have been recorded compared with women with normal bone density (Penoni et al., 2017).

In an in vitro study, where gingival fibroblasts were stimulated with IL‐1β to produce different MMPs (including MMP‐13), progesterone seemed to reduce their production (Collazos et al., 2015). Thus, hormonal effects on the downregulation of MMP‐13 in gingiva are possible, but how this does happen in vivo is not clear. In squamous cell carcinoma of the mouse skin, the effect of estrogen in the regulation of MMP‐13 inhibitor treatment was distinct so that older female mice had higher levels of MMP‐13 and lower levels of estradiol resulting in more effective MMP‐13 inhibitor treatment; if ovariectomy was made in young females, or 17β‐estradiol supplemented in older females, the effectiveness of the treatment was compromised (Meides et al., 2014). In postmenopausal women with CP, the expression of estrogen receptors in the gingiva is significantly reduced when compared with postmenopausal women with healthy periodontium (Karthik, Arun, Sudarsan, Talwar, & James, 2009). Some polymorphisms in the genes for MMP‐12 and MMP‐13 seem to be related to epithelial ovarian carcinoma (Li et al., 2009). In cervical cancer, MMP‐13 gene seems to be downregulated (Vazquez‐Ortiz et al., 2005). These results indeed speak for hormonal effects on the MMP‐13 metabolism.

In a previous study, MMP‐13 in saliva was higher in localized periodontitis but lower again in generalized periodontitis in a very similar range of values as seen in our present investigation (Gursoy et al., 2013). More recently, MMP‐8 and MMP‐13 in saliva increased with periodontitis disease progression and decreased after its nonsurgical treatment (Ozcan, Saygun, Serdar, Bengi, & Kantarci, 2016), but the concentration ranges were mostly higher for MMP‐13 and much lower for MMP‐8, than here reported. Thus, no direct comparisons can be made with our results. MMP‐13 was almost not detected in serum whereas MMP‐8, MMP‐9, and TIMP‐1 values in serum were very similar between the groups and in line with results from other studies (Turkoglu et al., 2014). The different results in different studies are probably due to different classifications for periodontitis (Savage, Eaton, Moles, & Needleman, 2009) and also because of different study population characteristics. The different immune tests (IFMA and ELISA) here used for the biomarkers have been shown to give similar and corresponding results, however (Nieminen et al., 2015).

No significant differences were found between the study groups in smoking habits, so this variable cannot be considered a relevant explanatory factor for the results obtained. The determination of smoking habits here used was a simple no/yes answer including smokers, ex‐smokers, and snuff users in the “yes” group. This gave a broader spectrum than only using pack‐years of smoking. Nonetheless, smoking is known to reduce the concentrations of biomarkers in saliva and other oral fluids, including MMPs and neutrophil elastase (Sorsa et al., 2016).

Finally, to sum up, our study hypothesis was not confirmed, because no significant difference was detected between the groups regarding MMP‐8, MMP‐9, and TIMP‐1 levels in saliva and serum. The salivary MMP‐13, on the other hand, was higher in the periodontally healthy group (based on the criteria here used) but, at the same time, associated with higher CAL recordings and appeared to link to female gender. This result is interesting in the view that this molecule was first detected in breast cancer samples and that its metabolism may reflect hormonal associations. The main weakness of our study is that we do not have data from the hormonal status of the patients. Nevertheless, the result opens up new prospects for hypothesis generation.

## CONFLICT OF INTEREST

The authors declare no conflicts of interest.
